# Supplementary material for: Abscisic Acid—Defensive Player in Flax Response to Fusarium culmorum Infection
Source: Molecules. 2022 Apr 29;27(9):2833. doi: 10.3390/molecules27092833 (PMC9105474; doi:10.3390/molecules27092833)

Supplementary File S1. The roots of the flax plants are growing through the medium and after exposition to *F. culmorum* are in direct contact with the mycelium.

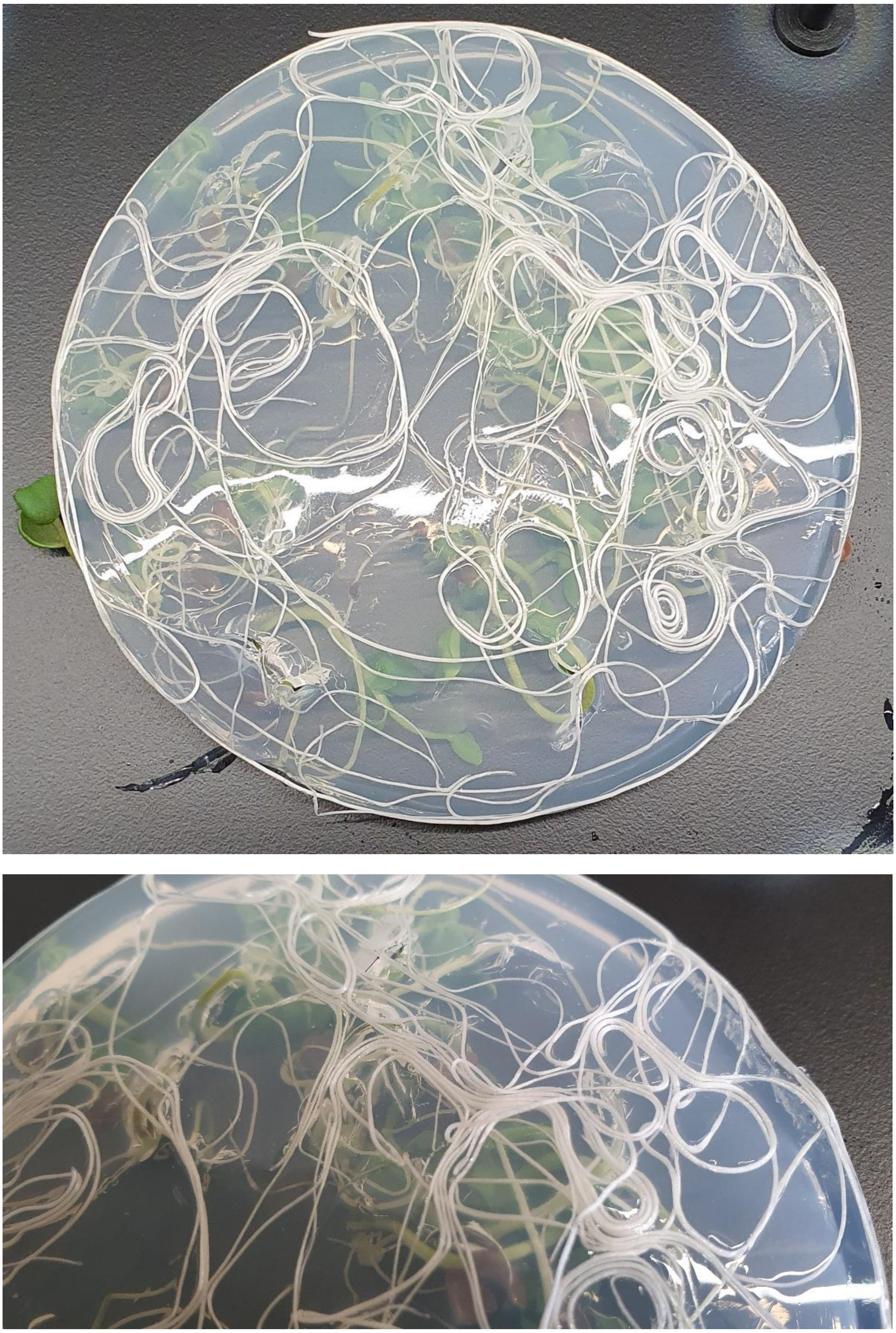

Supplement: Supplementary file 1 [file molecules-27-02833-s001.zip › Supplementary File S1.pdf]
