# Supplementary material for: Abscisic Acid—Defensive Player in Flax Response to Fusarium culmorum Infection
Source: Molecules. 2022 Apr 29;27(9):2833. doi: 10.3390/molecules27092833 (PMC9105474; doi:10.3390/molecules27092833)
Supplement: Supplementary file 1 [file molecules-27-02833-s001.zip › Supplementary File S2.pdf]

Supplementary File S2. List of primers used in the real-time RT-PCR.

- Terpenoid backbone biosynthesis gene primers

GENE	FWD PRIMER	REV PRIMER
ACTIN	CCGGTG TTATGG TTGGAA	TGTAGA AAGTGT GATGCC AAA
DXS	CGCTCC TACTCA ATCTTA CAC	CCGACA TCAAAG CACCT
DXR	ACTATT TGTTCG GAGCTG ATTA	GCCAGG ACATGG TGTAGA
HMGS	GCAAGG TTGGGC TTGTAA	GTGTGT ACTGAT AGTGCG G
HMGR	CCACCA AAGATT CAACAC TC	GACGTC ATCGGC ATCTC
FPPS	AGTATA AGACAG CATACT ACT	AGCAGT CCAGAT AATCAT CC
GPPS	CTTACC TATACC AAATAG TGTTCC G	TTTAAT GACTTA ATTCCA CGTCTT GT

- Carotenoid biosynthesis gene primers

GENE	FWD PRIMER	REV PRIMER
PSY	AGGGAT GAGAAT GGACTT GAA	AGCATT ATAGAC GCTCTC AG
PDS	GAGGCT CAAGAT GGTCTT AC	AAGAAA TCGGTT CAAGGC TA
Z-ISO	CACATA CACTGT GGATGG G	CTACAC TCGTCC TCTCCT TCA
ZCD	CCAGAT GCAGAC TTCTCG	AGAACC TGCTTT GATACT CTATT
CRTISO	TTTGAG CATTCC CACAAT ACT	GCCAAT AATCTC ATCAGC AAC
LCYB	GCCTTA CGGAAG GGTCAA TA	ATCGTA ACACCA TCGTTA CAT
LCE	GCAACT AATGGA CACAGT ATTGTA	CTCAAC CTCCAC ACCATA AG
βHY	GCGATT GGCCTA TTGTCA T	GCAGCT ACTTTG CGGAAA TAA
LUT1	GTCGCT GCACAA GAAGTA T	GAATTC AACGCG TCGAAA TTA
LUT5	ACTTTG GGCCAA ATGTCA	TGATGT TCAAGT GGGCTG
VDE	GCTCGA GAGAGC AGCTA	CTTGTC CTTCTA TCTCTT CAACC
ZEP	CTGCTT TGGAAG CCATTG	CGAGTA ACCGGA AGACC

- ABA and apocarotenoid biosynthesis gene primers

GENE	FWD PRIMER	REV PRIMER
CCD1	TGCATG ACTTTG CCATTA CTG	TTTGCA TATCGA GGTAAG ACAC
CCD7	CCAGGG TGATGT TTGGG	ATATCC TCGGCA TTGCAG
CCD8	AACGAA CGGAAG GTGAT	GAACTT GTAAAG CGGCGT C
NCED3	AGAAGC CGTACC TCAAG	TGCTGA TCCGGA ATCACC A
NCED6	CTGTCG ATACAC TCGAAC CA	CGTACT GTCTAA CAACCC G

ABA2	ATCGAC ATCCTC GTCAAC	GATGGG ATCATC ACTCTT GC
AAO3	GTGGAC GTTGAT GTGGAG	GAGTAA GCCAAC AGTGGT TAT

- Tocopherol biosynthesis gene primers

GENE	FWD PRIMER	REV PRIMER
GGR	GAAGTC CTCGAT GCTTAC C	TGCCGT CGTACT CAGTAT
VTE2	CCTTCC ATTGGC ATCCG	TGGCAG ATTGAT TGAATA GGC
VTE3	GCTTAT CATGGG ATGTTC TGTCA	CATAGT ATGTTG CAGCCA TTG
VTE1	GGGAAG TAGGCA TGAGC	TGTCTT TACAGT CTCCGC A
VTE4	ATGATA ATAGTG ACATGG TGCC	TATCCT GAAGAT TGAGGG ATTGG

- Sterol biosynthesis gene primers

GENE	FWD PRIMER	REV PRIMER
SQS	TATAAA CGAGAT ACCTAA GTCTCG C	ATATGT ATCAAA GCATTA GTGACC A
SQE	TCATAA TGGGCG CTTCAT AC	TGTCTG CAGTTC TCCGT
CAS	GTAATC AATAAC GATGTC ACCGA	CCACTT GAAGCA CAAAGA TACT
SMT1	TGGTCT GAAACC TGGACA	GTCTAC TCCTGC AAGACG
SMO2	GAGTAT GCTACT CCATTT GGACTA	AATGCG CTTCAA CTGTTT
STE1	ACCTTC ATGCAA CTCATC A	CAAGAA AGCTAT ATGGGT CCTG

- Callose biosynthesis gene primers

GENE	FWD PRIMER	REV PRIMER
PMR4	AAGGCG ATAGAA CTCGG	TCCAGA TGGATT AAATAC AAACGG A

- ROS processing gene primers

GENE	FWD PRIMER	REV PRIMER
SOD Cu	CCACTG TAACTG GAAACA TCT	GGTGTT CATCCT CAGGAG CA
SOD Mn	CTGAAG GTGCTG CACTC	CATGCT CCCAGA CATCAA T
SOD Fe	ACCATG AATTCT TCTGGG AATC	CACCCG GAACCA AACTG
CATALASE	TGCAAT CGTGGT TCCTGG TGTTTA	GGTGAT TGTTGT GATGAG CACACT
APx	GGTGGT CACACT TTGGG	GAAGAG CCTTGT CACTCG
NADPHOXD	CATCAC TTCTGC TCCCG	TTGTTG TTTCCC TGAAAT CCAT
NADPHOXF	GGAAAT GTGCTC ACGTTG	GAACGC TAAGGT AGTCAT CC

- Callose biosynthesis gene primers

GENE	FWD PRIMER	REV PRIMER

CALS1	GCTTCCATGTGGTTCTTAGT	CTCCCAGCTCTTATTCGC
CALS2	CCTCTTCCATGTGGTTCTTAG	TTATTTGCAGGCACACCG
CALS3	AGAGCAAGATAAAGACGACC	TATTGAGCAGAGATGTTTACAGG
CALS4	CTTTACTTCACGGAGGTGC	GCCCGAAGATTTCATAGACGA
