# Supplementary material for: Abscisic Acid—Defensive Player in Flax Response to Fusarium culmorum Infection
Source: Molecules. 2022 Apr 29;27(9):2833. doi: 10.3390/molecules27092833 (PMC9105474; doi:10.3390/molecules27092833)
Supplement: Supplementary file 1 [file molecules-27-02833-s001.zip › Supplementary File S3.pdf]

	DXS		DXR		FPPS		GPPS		HMGR		HMGS		IDI		MVK	
x-fold of the control																
6h	0.898482	0.078865	1.194287	0.291636	0.649359	0.162003	1.222801	0.09527	0.800763	0.278749	1.159539	0.167307	0.908801	0.424987	0.959923	0.248682
12h	0.65142	0.114601	2.665787	0.776789	0.942582	0.272886	1.094497	0.103607	1.083155	0.157618	0.851576	0.128477	1.601536	0.303709	1.265483	0.234728
24h	0.569029	0.062943	2.492611	0.362474	0.757737	0.055698	2.506598	0.06121	0.908211	0.056756	0.855756	0.035852	1.743217	0.443787	0.960911	0.18666
36h	0.511336	0.11787	1.2427	0.125516	0.233063	0.074681	2.360127	0.399684	0.957867	0.098746	0.748411	0.109235	2.116138	0.477597	1.74558	0.356701
48h	0.805484	0.144167	3.231821	0.742321	0.396382	0.036457	1.518188	0.009746	1.240218	0.130274	0.842052	0.002321	2.359854	0.119127	1.126042	0.47229

	GGR		GTM		VTE2		VTE3		VTE1		VTE4	
6h	1.208608	0.274309	1.555218	1.155493	0.957492	0.155949	1.214173	0.12493	1.10311	0.106421	0.964944	0.145181
12h	0.94478	0.066454	1.49859	0.163417	1.255539	0.185293	0.737969	0.062956	1.716009	0.103287	1.49859	0.163417
24h	0.621297	0.033522	1.469126	0.132867	1.546422	0.079681	1.023019	0.112223	1.307473	0.319874	1.469126	0.132867
36h	0.174993	0.020328	0.622966	0.331927	0.453257	0.111783	0.25967	0.020015	0.948869	0.37813	0.977707	0.046201
48h	0.874877	0.060775	0.798376	0.688835	2.345858	0.362268	0.727507	0.258767	1.719471	0.053409	1.338998	0.07572

	βHY (CRTZ)		CRTISO		LCYB		LCYE		LUT1		LUT5		PDS		PSY		VDE		ZDS		ZEP		Z-ISO	
6h	1.312046	0.20411	0.874207	0.239693	0.822261	0.098814	1.457602	0.183097	1.379587	0.207949	1.064872	0.088638	1.119917	0.065028	1.144904	0.187301	1.072348	0.041391	0.765872	0.268127	0.966842	0.134875	1.011535	0.030398
12h	2.260227	0.231784	1.046293	0.151253	0.658113	0.048102	0.808957	0.140563	0.769713	0.168501	0.896198	0.126885	1.011497	0.055482	1.067964	0.123596	0.767465	0.147702	1.552894	0.144521	0.868358	0.152995	1.652846	0.055622
24h	2.098966	0.184076	1.781907	0.208362	0.542171	0.075754	0.622145	0.103811	1.121898	0.289449	0.725935	0.030902	0.887958	0.170345	2.743568	0.262471	0.905394	0.0667	0.990279	0.071589	0.795829	0.156514	1.250586	0.166522
36h	3.64055	0.335893	2.371574	0.373451	0.31433	0.030225	0.267775	0.038703	0.456049	0.061308	0.384792	0.035464	0.459226	0.045679	0.943052	0.295944	1.084375	0.099775	0.536302	0.075832	0.516577	0.075873	1.20184	0.278602
48h	3.878237	0.344359	2.711182	0.417605	1.216699	0.342313	0.658706	0.020965	1.46163	0.256332	1.377137	0.004477	1.158354	0.175595	3.633638	0.35349	0.795601	0.13374	1.755972	0.14202	0.836213	0.034734	3.024133	0.724303

	ABA2		AAO3		CCD1		CCD7		CCD8		NCED3		NCED6	
6h	0.969875	0.119972	0.862999	0.09684	0.94658	0.163332	0.574386	0.066009	0.583631	0.042149	2.752706	0.936036	3.302669	0.688377
12h	0.741645	0.049394	2.018131	0.188476	0.803078	0.081582	0.287193	0.009969	0.614303	0.038674	4.590688	0.234614	8.231913	3.288739
24h	0.6558	0.049676	1.940416	0.182607	0.857013	0.093828	0.222597	0.010524	0.420911	0.05569	5.253014	0.55043	6.293971	1.363887
36h	0.634404	0.184088	3.792924	0.09264	0.974946	0.102428	0.132434	0.061148	0.600531	0.033229	10.89347	1.320234	22.66279	4.109993
48h	0.972278	0.218786	2.268457	0.109596	0.929402	0.111424	0.174876	0.049155	0.422385	0.048667	8.650118	1.235515	12.73505	1.949512

	CAS		SMO2		SMT1		SMT2		SQE		SQS		STE1		P450		SQE	
6h	0.856084	0.076259	0.797881	0.057354	0.756517	0.169961	1.006213	0.161888	0.651423	0.068175	0.634298	0.340720	0.042553	0.002128	1.215637	0.368946	0.651423	0.068175
12h	1.301082	0.283951	0.745663	0.053861	0.762196	0.207075	1.085407	0.20262	0.68014	0.124337	0.704187	0.303044	1.718223	0.207548	2.835273	0.396416	0.68014	0.124337
24h	0.497868	0.038232	0.603737	0.079889	0.707403	0.09233	1.490243	0.303967	0.573634	0.113558	0.240908	0.019170	0.601124	0.058018	2.700975	0.526508	0.573634	0.113558
36h	0.434405	0.054497	0.423436	0.039288	0.383769	0.029693	0.725053	0.085243	0.628123	0.086576	0.587492	0.111777	0.728061	0.313035	2.098659	0.039926	0.628123	0.086576
48h	0.548824	0.097846	0.361779	0.021205	0.687624	0.150986	0.738853	0.037534	0.577615	0.019446	0.724714	0.201150	1.066391	0.056669	9.1234	1.75472	0.577615	0.019446

Supplementary File S3. RQs of terpenoid pathway biosynthesis genes (means and sd (in gray)).
