# Supplementary material for: Abscisic Acid—Defensive Player in Flax Response to Fusarium culmorum Infection
Source: Molecules. 2022 Apr 29;27(9):2833. doi: 10.3390/molecules27092833 (PMC9105474; doi:10.3390/molecules27092833)
Supplement: Supplementary file 1 [file molecules-27-02833-s001.zip › Supplementary File S4.pdf]

x-fold of the control

	6h	12h	24h	36h	48h
squalene	0.880319	0.883879	0.333929	2.095436	1.409887
campesterol	0.81505	1.128253	0.973257	1.041506	1.077503
stigmasterol	0.74599	0.899437	0.934694	1.078362	1.058983
β-sitosterol	0.81714	1.055712	1.006616	1.060432	1.088588
sitostanol	0.569806	0.944616	1.172846	1.107517	0.990092
avenasterol	0.569567	0.823506	1.167515	1.098648	1.36974
neoxanthin	0.827756	1.15842	1.084446	1.069807	0.842089
violaxanthin	0.838569	1.240807	1.101486	1.087136	0.903072
lutein	0.85406	1.150562	1.089812	1.067631	0.86999
chlorophyll b	0.862415	1.13238	1.080505	1.027036	0.854174
chlorophyll a	0.863798	1.174329	1.063973	1.004485	0.828228
β-carotene	0.87255	1.226519	1.073992	0.986127	0.905171
tocopherol	0.868726	1.171854	1.112807	1.017141	0.978179
ABA	3.373088	7.599848	5.845432	4.944583	6.720674

Supplementary File S4. Levels of the terpenoid metabolites in flax after *F. culmorum* infection (x-fold of the control).
