# Supplementary material for: Abscisic Acid—Defensive Player in Flax Response to Fusarium culmorum Infection
Source: Molecules. 2022 Apr 29;27(9):2833. doi: 10.3390/molecules27092833 (PMC9105474; doi:10.3390/molecules27092833)
Supplement: Supplementary file 1 [file molecules-27-02833-s001.zip › Supplementary File S5.pdf]

Supplementary File S5. Influence of carotenoids on *F. culmorum* growth.

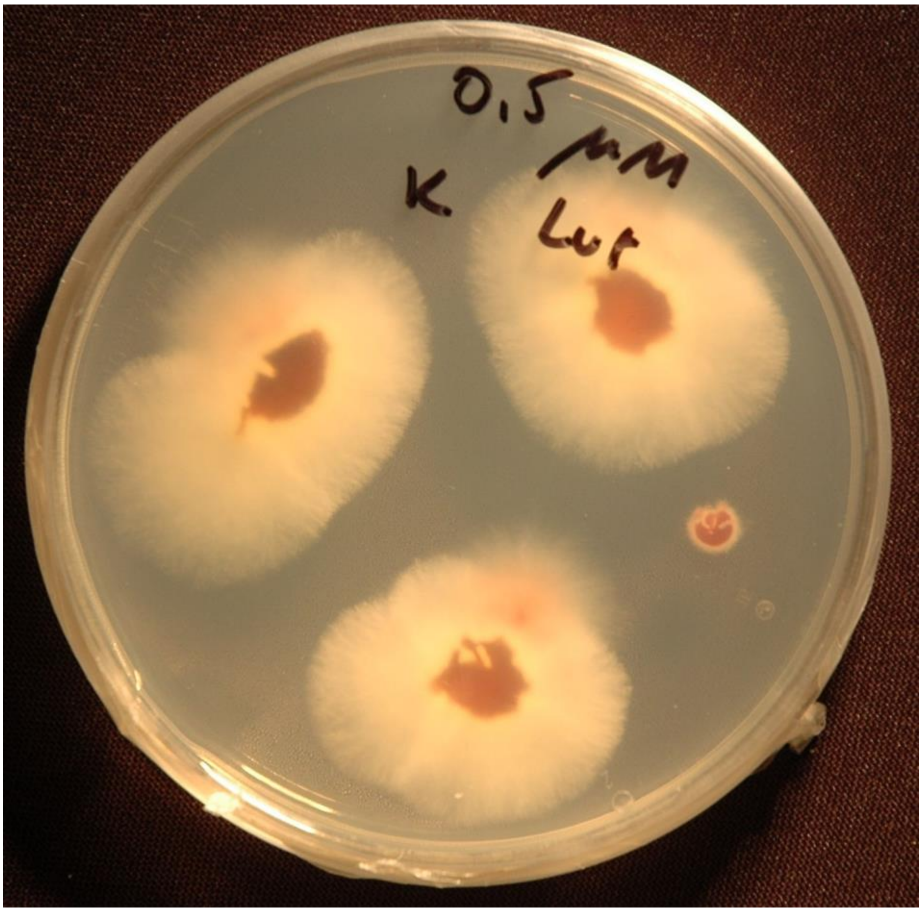

F. culmorum – control (for carotenoids)

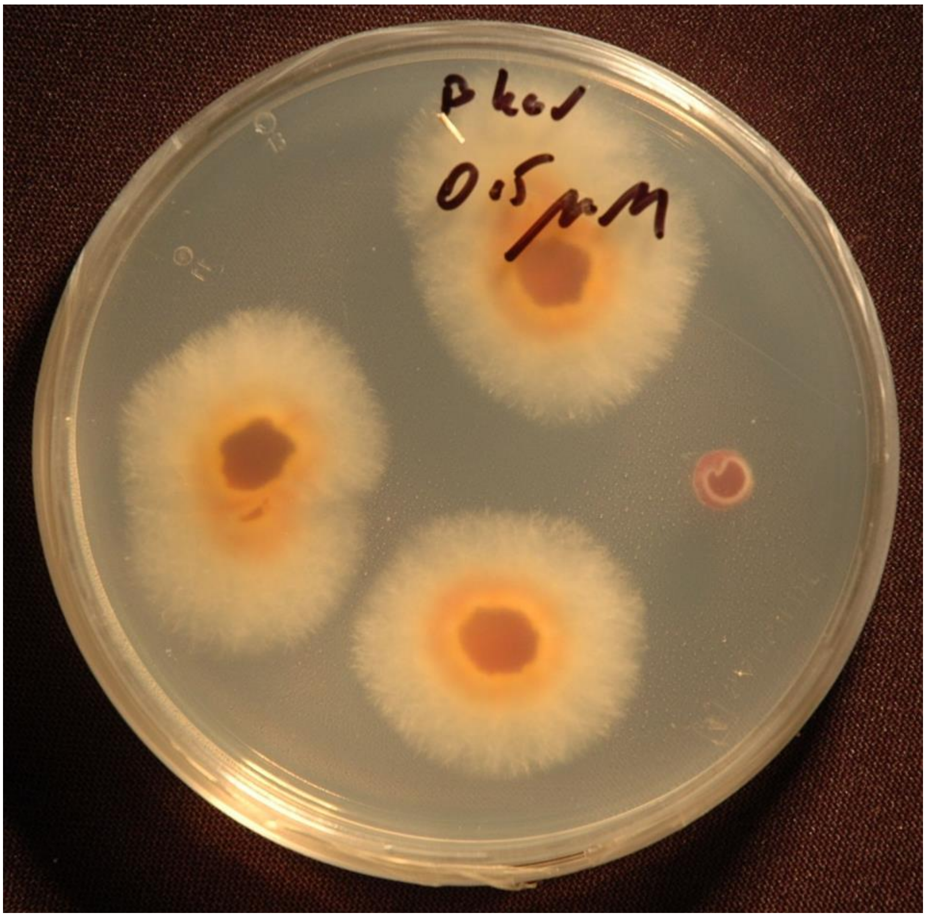

F. culmorum + 0.5 µM β-carotene

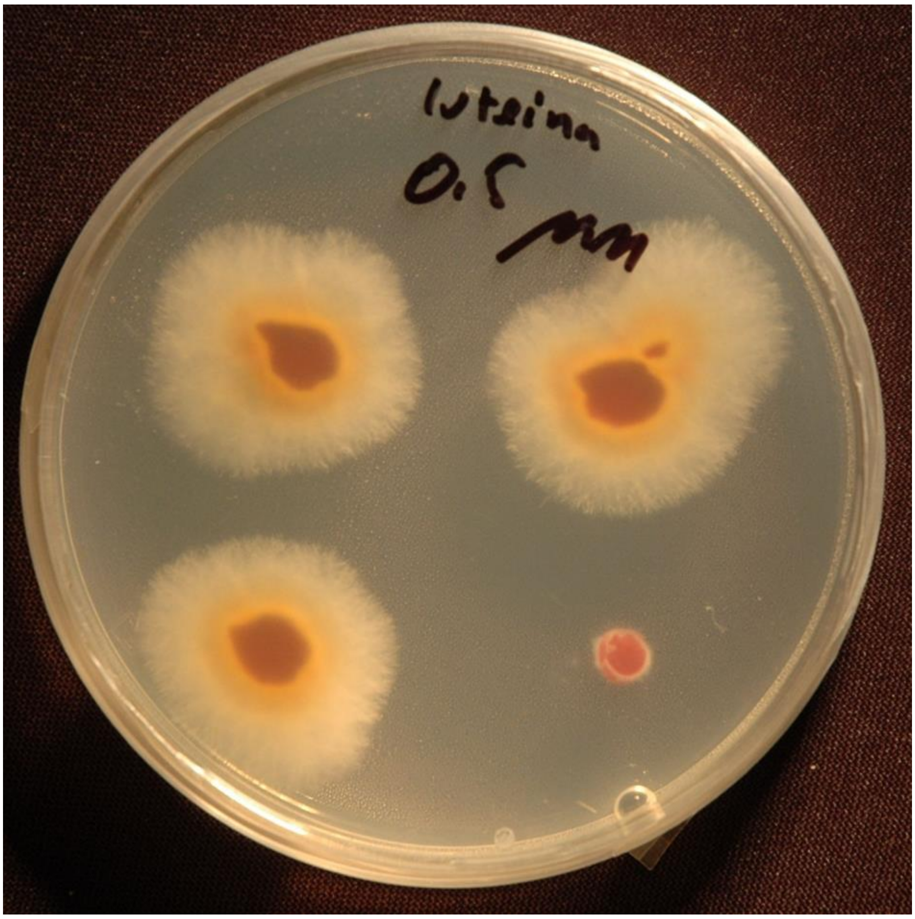

F. culmorum + 0.5 µM lutein

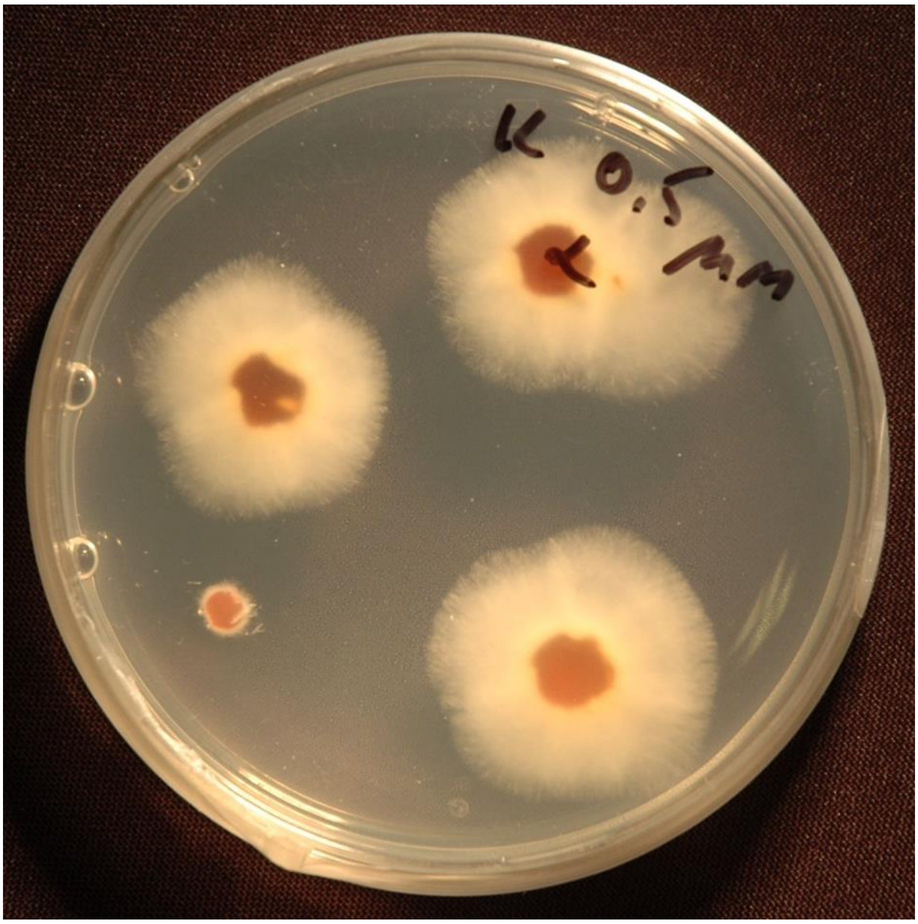

F. culmorum – control (for tocopherols)

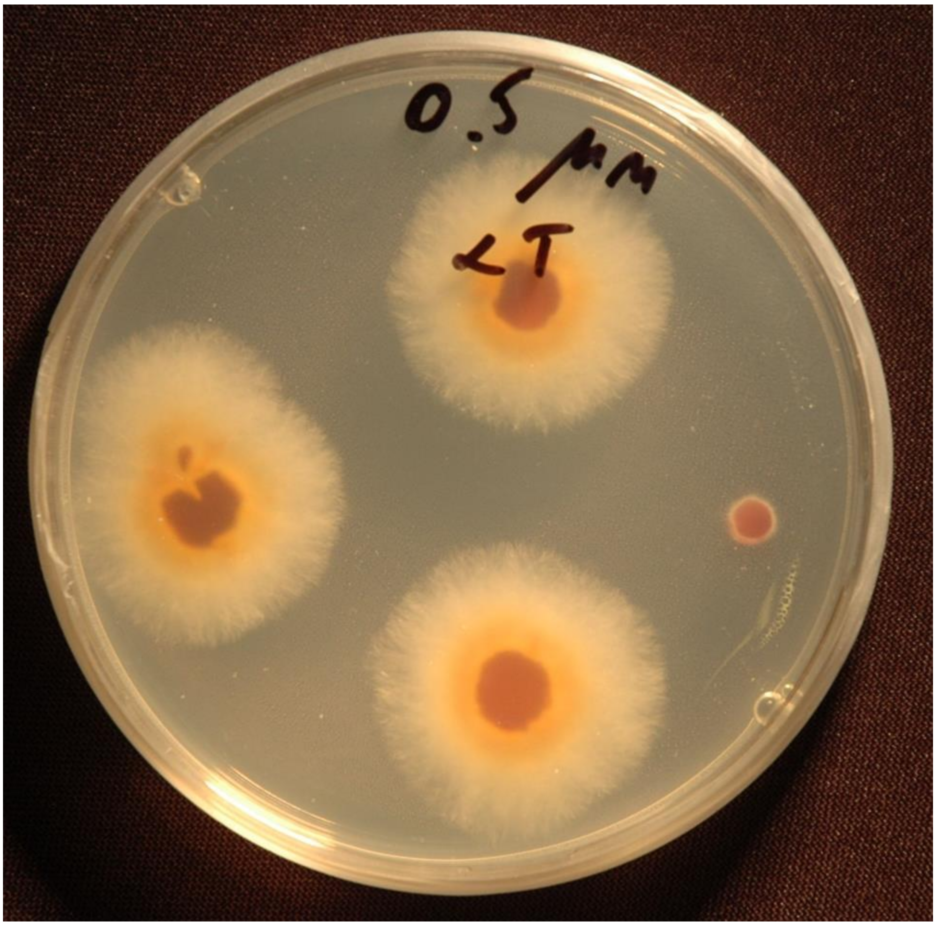

F. culmorum + 0.5 µM α-tocopherol

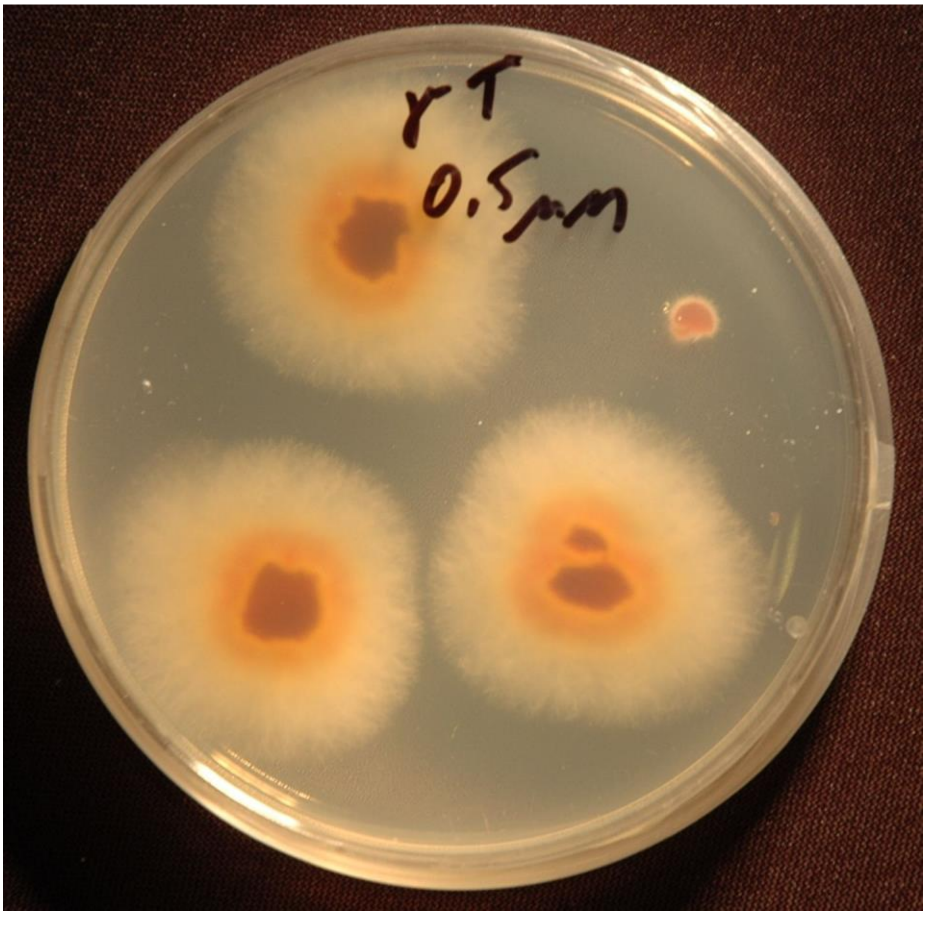

F. culmorum + 0.5 µM γ-tocopherol
