# Supplementary material for: Abscisic Acid—Defensive Player in Flax Response to Fusarium culmorum Infection
Source: Molecules. 2022 Apr 29;27(9):2833. doi: 10.3390/molecules27092833 (PMC9105474; doi:10.3390/molecules27092833)
Supplement: Supplementary file 1 [file molecules-27-02833-s001.zip › Supplementary File S6.pdf]

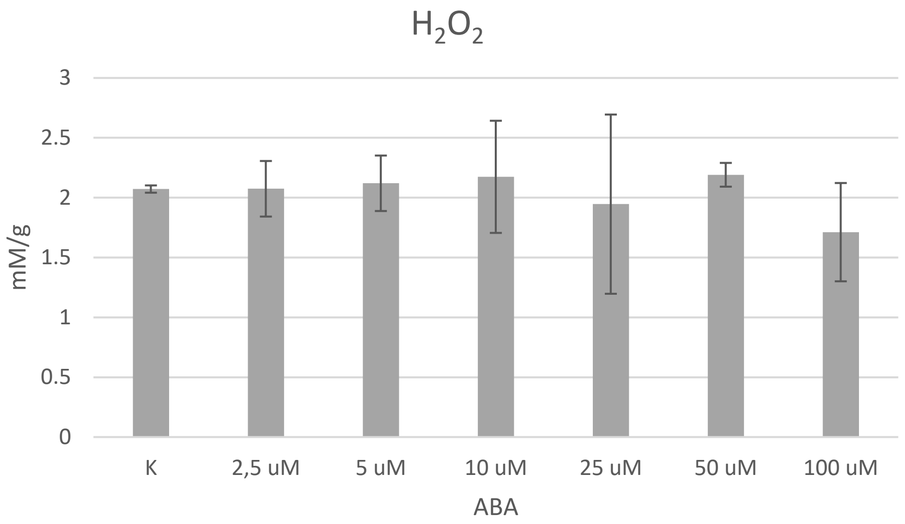
H2O2
mM/g
3
2.5
2
1.5
1
0.5
0
K
2,5 uM
5 uM
10 uM
25 uM
50 uM
100 uM
ABA

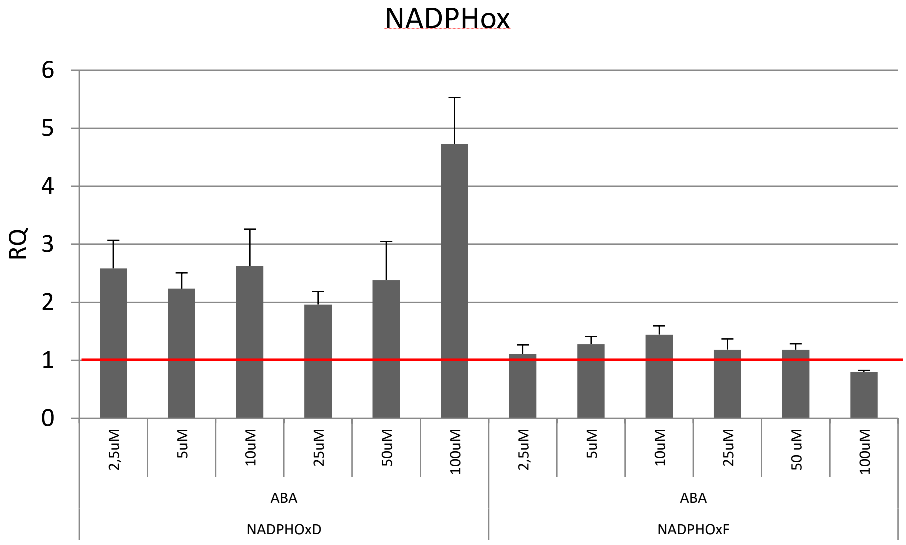
NADPHox
RQ
6
5
4
3
2
1
0
2,5uM
5uM
10uM
25uM
50uM
100uM
2,5uM
5uM
10uM
25uM
50 uM
100uM
ABA
ABA
NADPHOxD
NADPHOxF

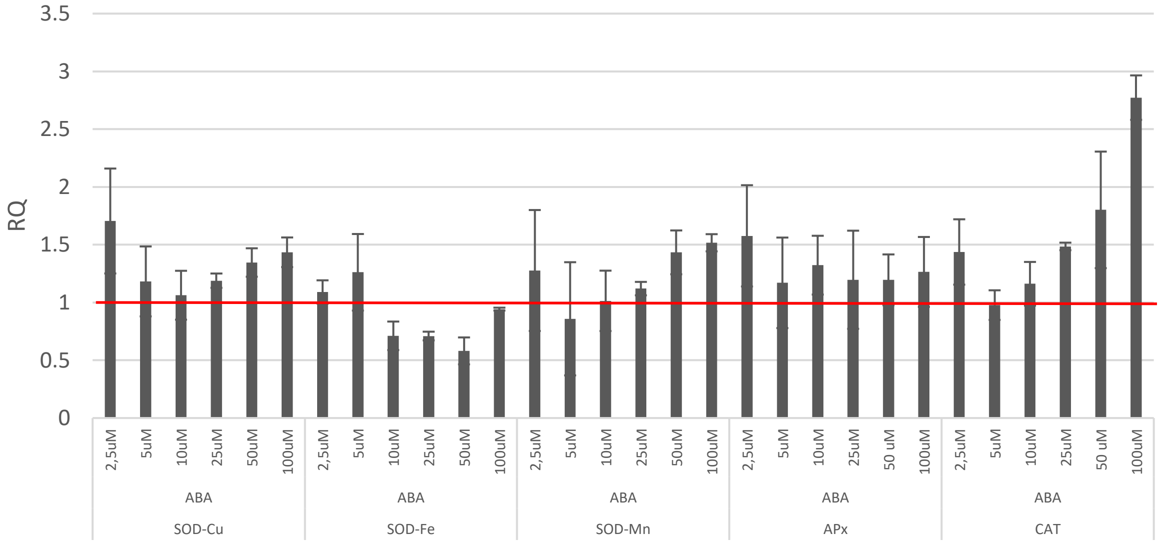
RQ
3.5
3
2.5
2
1.5
1
0.5
0
2,5uM
5uM
10uM
25uM
50uM
100uM
2,5uM
5uM
10uM
25uM
50uM
100uM
2,5uM
5uM
10uM
25uM
50uM
100uM
2,5uM
5uM
10uM
25uM
50 uM
100uM
2,5uM
5uM
10uM
25uM
50 uM
100uM
ABA
ABA
ABA
ABA
ABA
SOD-Cu
SOD-Fe
SOD-Mn
APx
CAT

Supplementary File S6. The content of H_2O_2 in flax seedlings sprayed with different concentrations of ABA (A); transcript levels of NADPHOxD and NADPHOxF in flax seedlings sprayed with different concentrations of ABA presented as relative quantification (RQ) compared to control (RQ = 1 marked with red line) with actin used as reference gene (B); transcript levels of superoxide dismutase (SOD-Cu, SOD-Fe, SOD-Mn), ascorbate peroxidase (APx) and catalase (CAT) genes in flax seedlings sprayed with different concentrations of ABA presented as relative quantification (RQ) compared to control (RQ = 1 marked with red line) with actin used as reference gene (C).
